# 2-Hy­droxy­ethyl­ammonium [2-(2,6-di­chloro­anilino)phen­yl]acetate monohydrate

**DOI:** 10.1107/S2414314622004412

**Published:** 2022-04-28

**Authors:** Nodira Obidova, Jamshid Ashurov, Lidiya Izotova, Bakhtiyar Ibragimov

**Affiliations:** a Institute of Bioorganic Chemistry, UzAS, M. Ulugbek Str., 83, 100125, Tashkent, Uzbekistan; Benemérita Universidad Autónoma de Puebla, México

**Keywords:** crystal structure, diclofenac, complex, hydrogen bonding.

## Abstract

In the solid-state structure of the title compound derived from diclofenac, C_2_H_8_NO^+^·C_14_H_10_Cl_2_NO_2_^−^·H_2_O, the asymmetric unit contains one cation, one anion and a water mol­ecule, all in general positions. A complex network of hydrogen bonds is present in the crystal structure.

## Abstract 2

A new organic salt of diclofenac, [2-(2,6-di­chloro­anilino)phen­yl]acetate, with mono­ethano­lammonium and water has been obtained, and its structure has been established by X-ray analysis.

## Graphical abstract

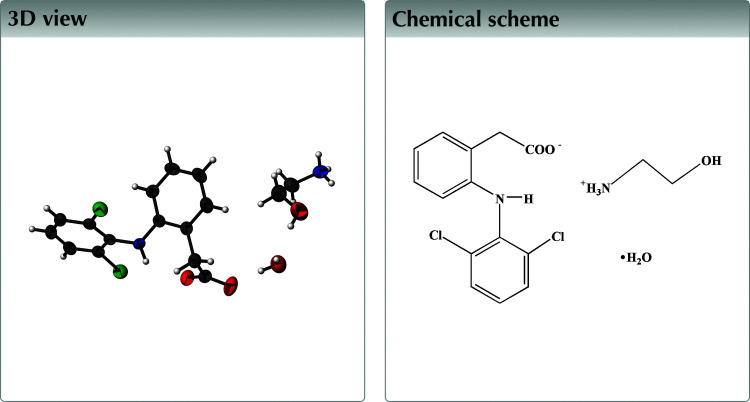

## Structure description

The pharmaceutical diclofenac (D) is widely used as a non-steroidal anti-inflammatory drug, to treat pain and inflammatory diseases (Skoutakis *et al.*, 1988 ▸; Moser *et al.*, 1990 ▸). The Cambridge Structural Database (CSD version 5.42, last update February 2021; Groom *et al.*, 2016 ▸) includes crystallographic data for 50 entries with the term ‘diclofenac’. Among them, there are 21 entries where diclofenac is present in the form of a salt, and in three entries, diclofenac forms salts with aliphatic amines: with (*R*) and (*S*)-phenyl­ethyl­ammonium (Lemmerer *et al.*, 2010 ▸), with diethyl ammonium (Castellari *et al.*, 2001 ▸) and with tris­(2-ammonio­eth­yl)amine (Lynch *et al.*, 2003 ▸). In this article, we present another complex in the form of a diclofenac salt with an amino-containing compound, namely mono­ethano­lamine. Ethano­lamine is always present in significant qu­anti­ties in the human and animal body with a complete protein diet. Its formation occurs during the deca­rboxylation of serine, and in one of the metabolic variants, it turns into glycine (the simplest aliphatic amino acid; Wishart *et al.*, 2007 ▸). In addition, mono­ethano­lamine is used in some cosmetic products (Knaak *et al.*, 1997 ▸). Therefore, the inter­action of these compounds seems to be inter­esting for investigation.

**Figure 1:**
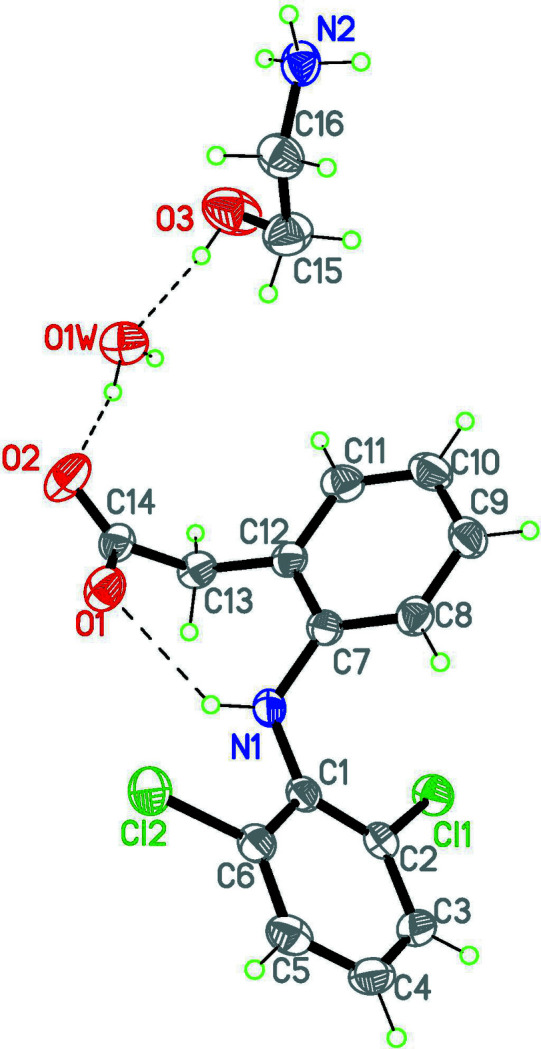
Perspective view of the title compound with the atom-numbering scheme. Displacement ellipsoids are drawn at 40% probability level. The dashed lines represent hydrogen bonds within the asymmetric unit.

The crystal structure of the title compound has one mono­ethano­lamine (MEA) cation, one 2-(2,6-di­chloro­anilino)phenyl acetic acid or diclofenac (D) anion, and one water mol­ecule in the asymmetric unit, and crystallizes in space group *P*2_1_/*c* (Fig. 1 ▸). The diclofenac anion is stabilized by one intra­molecular hydrogen bond between the amino group and atom O1 of the carb­oxy­lic group: N1—H1⋯O1 [2.884 (3) Å, 128.9°; see Table 1 ▸], which forms a seven-membered ring with graph-set notation *S*(7) (Etter, 1990 ▸). The dihedral angle between the two benzene rings in D is 60.2 (2)°.

**Figure 2:**
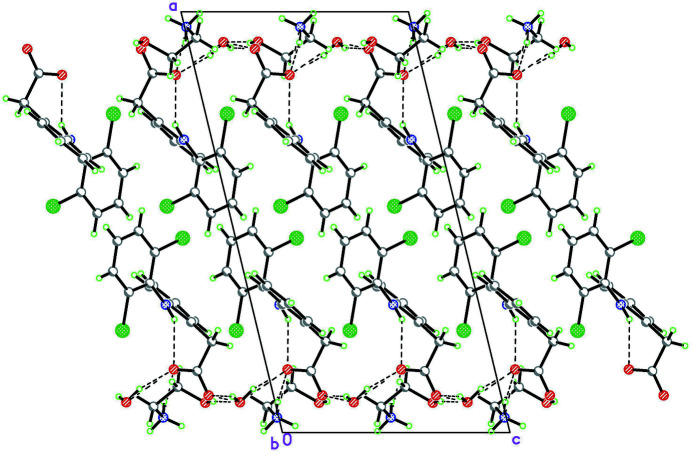
Packing diagram of the title compound, viewed down *b* axis. The hydrogen bonds are shown as dashed lines.

The ionic form of the title compound serves as a building block for the supra­molecular architecture. In the crystal, the building blocks form screw-like chains along the *b*-axis direction, due to the crystallographic twofold screw axis, *via* N2—H2*B*⋯O1*W* ^ii^ hydrogen bond [2.947 (4) Å, symmetry code: (ii) −*x*, *y* − 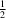 , −*z* + 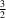 ; Fig. 2 ▸ and Table 1 ▸]. The chains are further consolidated into two-dimensional layers through N—H⋯O and O—H⋯O hydrogen bonds. These layers propagate parallel to the (100) plane, where the chains are related by the glide plane *c* [O1*W⋯*O1^iv^, symmetry code: (iv) *x*, −*y* + 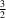 , *z* + 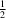 ; 2.809 (3) Å] and the inversion centre [N2... O2^i^, symmetry code: (i) −*x*, −*y* + 1, −*z* + 1, 2.811 (4) Å, Fig. 2 ▸]. The layers are linked by Y—*X*⋯*Cg π*–ring inter­actions, for C3—H3 and C7—Cl1 bonds, for which the *X*⋯*Cg* separations and *γ* angles range from 3.533 to 3.958 Å and from 25.03 to 28.79°.

**Figure 3:**
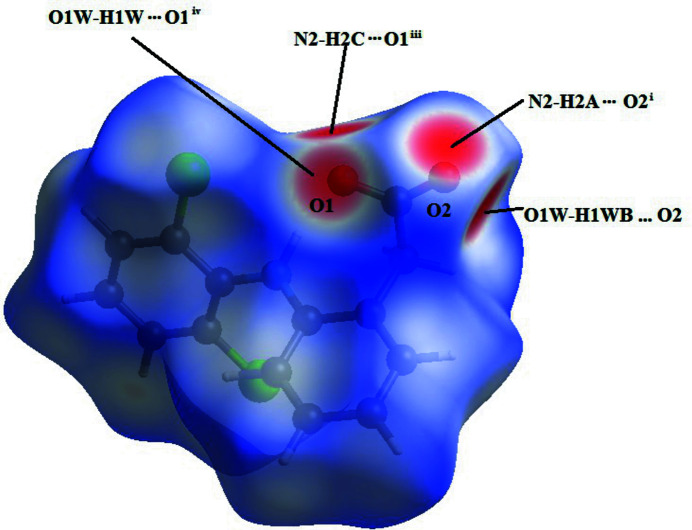
The Hirshfeld surface analysis indicates that the most important contributions to the crystal packing are from H⋯H (31.0%), H⋯C/C⋯H (26.3%) and H⋯Cl/Cl⋯H (25%) inter­actions.

**Figure 4:**
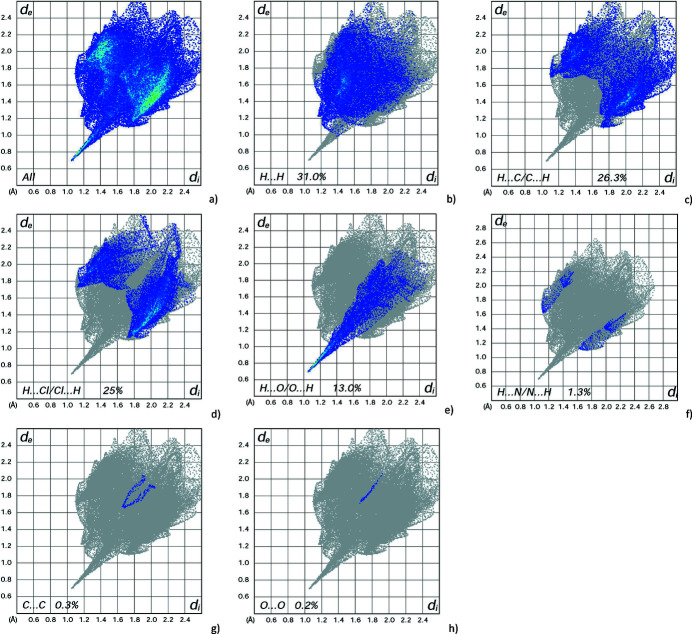
Full two-dimensional fingerprint plots for the title compound, showing all inter­actions (*a*), and delineated into (*b*) H⋯H, (*c*) H⋯C/C⋯H, (*d*) H⋯Cl/Cl⋯H, (*e*) H⋯O/O⋯H, (*f*) H⋯N/N⋯H, (*g*) C⋯C and (*h*) O⋯O inter­actions. The *d*_i_ and *d*_e_ values are the closest inter­nal and external distances (in Å) from a given point on the Hirshfeld surface depicted in Fig. 3 ▸.

In order to visualize the inter­molecular inter­actions in the crystal of the title compound, a Hirshfeld surface analysis was carried out using *Crystal Explorer 17.5* (Turner *et al.*, 2017 ▸). The Hirshfeld surface mapped over *d*_norm_ shows the expected bright-red spots near atoms O1 and O2, involved in the O—H⋯O and N—H⋯O hydrogen-bonding inter­actions (Fig. 3 ▸). Fingerprint plots (Fig. 4 ▸) reveal that H⋯H, H⋯C/C⋯H, H⋯Cl/Cl⋯H and H⋯O/O⋯H inter­actions make the greatest contributions to the surface contacts (Table 1 ▸), while H⋯N/N⋯H, C⋯C and O⋯O contacts are much less significant.

## Synthesis and crystallization

To a solution of 0.1 g (0.52 mmol) of D in 4 ml of ethanol, 32 µ*L* of mono­ethano­lamine was added. The mixture was kept in an ultrasonic bath (30 kHz) at 298 K for 5 min. The solution was then placed in a loosely closed bottle and kept at 298 K for 10 days. The precipitated prismatic crystals were selected for the single-crystal X-ray diffraction analysis.

## Refinement

Crystal data, data collection and structure refinement details are summarized in Table 2 ▸.

## Supplementary Material

Crystal structure: contains datablock(s) I. DOI: 10.1107/S2414314622004412/bh4068sup1.cif

Structure factors: contains datablock(s) I. DOI: 10.1107/S2414314622004412/bh4068Isup2.hkl

Supporting information file. DOI: 10.1107/S2414314622004412/bh4068Isup3.cml

CCDC reference: 2168795

Additional supporting information:  crystallographic information; 3D view; checkCIF report

## Figures and Tables

**Table 1:** Hydrogen-bond geometry (Å, °)

*D*—H⋯*A*	*D*—H	H⋯*A*	*D*⋯*A*	*D*—H⋯*A*
N1—H1⋯O1	0.86	2.27	2.884 (3)	129
N2—H2*A*⋯O2^i^	0.89	1.96	2.811 (4)	160
N2—H2*B*⋯O1*W* ^ii^	0.89	2.15	2.947 (4)	148
N2—H2*C*⋯O1^iii^	0.89	1.92	2.802 (3)	169
O3—H3*A*⋯O1*W*	0.82	1.96	2.770 (4)	168
O1*W*—H1*WB*⋯O2	0.85	1.87	2.690 (3)	161
O1*W*—H1*WA*⋯O1^iv^	0.85	2.00	2.809 (3)	158

Symmetry codes: (i) 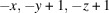 ; (ii) 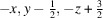 ; (iii) 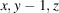 ; (iv) 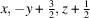 .

**Table 2:** Experimental details

Crystal data	
Chemical formula	C_2_H_8_NO^+^·C_14_H_10_Cl_2_NO_2_^−^·H_2_O
*M*_r_	375.24
Crystal system, space group	Monoclinic, *P*2_1_/*c*
Temperature (K)	293
*a*, *b*, *c* (Å)	19.1257 (10), 9.3864 (5), 10.0502 (6)
β (°)	103.546 (6)
*V* (Å^3^)	1754.05 (17)
*Z*	4
Radiation type	Cu *K*α
μ (mm^−1^)	3.53
Crystal size (mm)	0.31 × 0.28 × 0.1

Data collection	
Diffractometer	Agilent Technologies Xcalibur, Ruby
Absorption correction	Multi-scan (*CrysAlis PRO*; Agilent, 2014 ▸)
*T*_min_, *T*_max_	0.356, 1.000
No. of measured, independent and observed [*I* > 2σ(*I*)] reflections	12416, 3621, 2431
*R*_int_	0.078
(sin θ/λ)_max_ (Å^−1^)	0.631

Refinement	
*R*[*F* ^2^ > 2σ(*F* ^2^)], *wR*(*F* ^2^), *S*	0.051, 0.134, 1.01
No. of reflections	3621
No. of parameters	222
H-atom treatment	H-atom parameters constrained
Δρ_max_, Δρ_min_ (e Å^−3^)	0.40, −0.35

Computer programs: *CrysAlis PRO* (Agilent, 2014 ▸), *SHELXT2018/2* (Sheldrick, 2015*a* ▸), *SHELXL2018/3* (Sheldrick, 2015*b* ▸), *XP* (Siemens, 1994 ▸), *Mercury* (Macrae *et al.*, 2020 ▸) and *publCIF* (Westrip, 2010 ▸).

## full crystallographic data

### Crystal data

**Table:**

C_2_H_8_NO^+^·C_14_H_10_Cl_2_NO_2_^−^·H_2_O	*F*(000) = 784
*M**_r_* = 375.24	*D*_x_ = 1.421 Mg m^−^^3^
Monoclinic, *P*2_1_/*c*	Cu *K*α radiation, λ = 1.54184 Å
*a* = 19.1257 (10) Å	Cell parameters from 2166 reflections
*b* = 9.3864 (5) Å	θ = 4.7–73.9°
*c* = 10.0502 (6) Å	µ = 3.53 mm^−^^1^
β = 103.546 (6)°	*T* = 293 K
*V* = 1754.05 (17) Å^3^	Prism, colourless
*Z* = 4	0.31 × 0.28 × 0.1 mm

### Data collection

**Table:**

Agilent Technologies Xcalibur, Ruby diffractometer	2431 reflections with *I* > 2σ(*I*)
Radiation source: fine-focus sealed tube	*R*_int_ = 0.078
/w scans	θ_max_ = 76.5°, θ_min_ = 4.8°
Absorption correction: multi-scan (CrysAlis PRO; Agilent, 2014)	*h* = −23→24
*T*_min_ = 0.356, *T*_max_ = 1.000	*k* = −11→6
12416 measured reflections	*l* = −12→12
3621 independent reflections	

### Refinement

**Table:**

Refinement on *F*^2^	Primary atom site location: dual
Least-squares matrix: full	Secondary atom site location: difference Fourier map
*R*[*F*^2^ > 2σ(*F*^2^)] = 0.051	Hydrogen site location: mixed
*wR*(*F*^2^) = 0.134	H-atom parameters constrained
*S* = 1.01	*w* = 1/[σ^2^(*F*_o_^2^) + (0.0614*P*)^2^] where *P* = (*F*_o_^2^ + 2*F*_c_^2^)/3
3621 reflections	(Δ/σ)_max_ < 0.001
222 parameters	Δρ_max_ = 0.40 e Å^−^^3^
0 restraints	Δρ_min_ = −0.35 e Å^−^^3^

### Special details

**Table:**

Refinement. All hydrogen atoms were placed in idealized positions and refined as riding to their carrier atoms.

### Fractional atomic coordinates and isotropic or equivalent isotropic displacement parameters (Å^2^)

**Table:**

	*x*	*y*	*z*	*U*_iso_*/*U*_eq_	
Cl1	0.46202 (4)	0.95109 (8)	0.76764 (8)	0.0441 (2)	
Cl2	0.24250 (4)	1.20690 (9)	0.40514 (9)	0.0500 (2)	
O1	0.15000 (11)	0.9926 (2)	0.5897 (2)	0.0472 (6)	
O1W	0.07192 (13)	0.6316 (3)	0.8457 (2)	0.0558 (6)	
H1WA	0.103624	0.612215	0.917982	0.084*	
H1WB	0.085392	0.710654	0.818732	0.084*	
N1	0.30390 (13)	0.9959 (2)	0.6226 (3)	0.0379 (6)	
H1	0.269351	1.041181	0.645204	0.045*	
O2	0.08825 (11)	0.8748 (3)	0.7146 (2)	0.0592 (7)	
N2	0.03573 (14)	0.1808 (3)	0.4921 (3)	0.0446 (6)	
H2A	0.003371	0.148791	0.419647	0.054*	
H2B	0.015651	0.187499	0.563284	0.054*	
H2C	0.072670	0.120586	0.512028	0.054*	
O3	0.07091 (17)	0.3857 (3)	0.6928 (3)	0.0762 (9)	
H3A	0.073883	0.464400	0.729146	0.114*	
C2	0.42880 (15)	1.0634 (3)	0.6305 (3)	0.0327 (6)	
C14	0.14604 (15)	0.9162 (3)	0.6906 (3)	0.0371 (7)	
C1	0.35476 (15)	1.0754 (3)	0.5751 (3)	0.0322 (6)	
C12	0.26283 (14)	0.7823 (3)	0.7158 (3)	0.0342 (6)	
C7	0.30532 (15)	0.8463 (3)	0.6360 (3)	0.0342 (6)	
C8	0.34720 (15)	0.7613 (3)	0.5707 (3)	0.0382 (7)	
H8	0.374195	0.803323	0.515422	0.046*	
C6	0.33381 (16)	1.1786 (3)	0.4739 (3)	0.0359 (6)	
C13	0.21653 (15)	0.8708 (3)	0.7865 (3)	0.0383 (7)	
H13A	0.243062	0.955166	0.824815	0.046*	
H13B	0.206067	0.816317	0.861507	0.046*	
C9	0.34871 (16)	0.6146 (3)	0.5877 (3)	0.0447 (8)	
H9	0.377768	0.559121	0.546037	0.054*	
C5	0.38298 (18)	1.2604 (3)	0.4257 (3)	0.0430 (7)	
H5	0.366991	1.328721	0.358405	0.052*	
C4	0.45510 (18)	1.2404 (3)	0.4774 (3)	0.0459 (8)	
H4	0.488229	1.292653	0.442936	0.055*	
C3	0.47873 (16)	1.1423 (3)	0.5811 (3)	0.0408 (7)	
H3	0.527698	1.129421	0.617432	0.049*	
C11	0.26455 (17)	0.6344 (3)	0.7292 (3)	0.0436 (8)	
H11	0.236429	0.590723	0.781473	0.052*	
C10	0.30743 (18)	0.5508 (3)	0.6661 (3)	0.0488 (8)	
H10	0.308209	0.452307	0.676839	0.059*	
C16	0.06160 (19)	0.3234 (3)	0.4609 (4)	0.0515 (8)	
H16A	0.020800	0.385304	0.426179	0.062*	
H16B	0.088673	0.314490	0.390895	0.062*	
C15	0.1082 (2)	0.3869 (4)	0.5871 (4)	0.0593 (10)	
H15A	0.152356	0.332453	0.614785	0.071*	
H15B	0.120699	0.483999	0.568925	0.071*	

### Atomic displacement parameters (Å^2^)

**Table:**

	*U*^11^	*U*^22^	*U*^33^	*U*^12^	*U*^13^	*U*^23^
Cl1	0.0413 (4)	0.0418 (4)	0.0442 (4)	0.0030 (3)	0.0002 (3)	0.0044 (3)
Cl2	0.0424 (4)	0.0481 (5)	0.0552 (5)	0.0075 (3)	0.0025 (4)	0.0094 (4)
O1	0.0363 (12)	0.0534 (14)	0.0497 (13)	0.0053 (10)	0.0057 (10)	0.0195 (11)
O1W	0.0600 (15)	0.0558 (16)	0.0496 (15)	−0.0060 (12)	0.0087 (12)	0.0047 (11)
N1	0.0349 (13)	0.0309 (13)	0.0525 (15)	0.0053 (10)	0.0196 (12)	0.0055 (11)
O2	0.0325 (12)	0.0799 (18)	0.0625 (16)	−0.0032 (11)	0.0054 (11)	0.0235 (13)
N2	0.0409 (14)	0.0497 (16)	0.0404 (15)	0.0072 (12)	0.0037 (12)	−0.0030 (12)
O3	0.106 (2)	0.0641 (18)	0.0689 (18)	−0.0190 (17)	0.0403 (17)	−0.0214 (14)
C2	0.0376 (15)	0.0276 (14)	0.0331 (15)	0.0030 (12)	0.0087 (12)	−0.0018 (12)
C14	0.0317 (15)	0.0414 (17)	0.0364 (17)	0.0012 (13)	0.0043 (13)	−0.0003 (13)
C1	0.0351 (15)	0.0267 (14)	0.0347 (15)	0.0014 (11)	0.0080 (12)	−0.0029 (11)
C12	0.0283 (14)	0.0355 (16)	0.0351 (16)	0.0000 (12)	−0.0003 (12)	0.0050 (12)
C7	0.0300 (14)	0.0310 (15)	0.0377 (16)	0.0010 (11)	0.0002 (12)	0.0027 (12)
C8	0.0328 (15)	0.0415 (17)	0.0389 (17)	0.0001 (13)	0.0056 (13)	−0.0017 (13)
C6	0.0373 (15)	0.0322 (15)	0.0374 (16)	0.0013 (12)	0.0071 (13)	−0.0013 (12)
C13	0.0352 (15)	0.0428 (17)	0.0351 (16)	−0.0017 (13)	0.0044 (13)	0.0068 (13)
C9	0.0415 (17)	0.0377 (17)	0.0498 (19)	0.0045 (14)	0.0004 (15)	−0.0099 (15)
C5	0.057 (2)	0.0333 (16)	0.0410 (18)	−0.0019 (14)	0.0157 (16)	0.0027 (13)
C4	0.0494 (19)	0.0401 (18)	0.053 (2)	−0.0111 (15)	0.0226 (17)	−0.0036 (15)
C3	0.0346 (16)	0.0380 (17)	0.0505 (19)	−0.0056 (13)	0.0115 (14)	−0.0067 (14)
C11	0.0441 (18)	0.0387 (17)	0.0447 (19)	−0.0035 (14)	0.0039 (15)	0.0110 (14)
C10	0.052 (2)	0.0313 (16)	0.058 (2)	−0.0001 (14)	0.0025 (17)	0.0041 (15)
C16	0.059 (2)	0.0442 (19)	0.049 (2)	0.0028 (16)	0.0073 (17)	0.0046 (15)
C15	0.055 (2)	0.064 (2)	0.058 (2)	−0.0107 (18)	0.0104 (18)	−0.0099 (19)

### Geometric parameters (Å, º)

**Table:**

Cl1—C2	1.734 (3)	C7—C8	1.398 (4)
Cl2—C6	1.741 (3)	C8—C9	1.387 (4)
O1—C14	1.259 (3)	C8—H8	0.9300
O1W—H1WA	0.8501	C6—C5	1.387 (4)
O1W—H1WB	0.8504	C13—H13A	0.9700
N1—C1	1.396 (3)	C13—H13B	0.9700
N1—C7	1.410 (3)	C9—C10	1.377 (4)
N1—H1	0.8600	C9—H9	0.9300
O2—C14	1.248 (3)	C5—C4	1.368 (5)
N2—C16	1.486 (4)	C5—H5	0.9300
N2—H2A	0.8900	C4—C3	1.384 (4)
N2—H2B	0.8900	C4—H4	0.9300
N2—H2C	0.8900	C3—H3	0.9300
O3—C15	1.412 (4)	C11—C10	1.391 (5)
O3—H3A	0.8200	C11—H11	0.9300
C2—C3	1.389 (4)	C10—H10	0.9300
C2—C1	1.400 (4)	C16—C15	1.494 (5)
C14—C13	1.523 (4)	C16—H16A	0.9700
C1—C6	1.394 (4)	C16—H16B	0.9700
C12—C11	1.395 (4)	C15—H15A	0.9700
C12—C7	1.403 (4)	C15—H15B	0.9700
C12—C13	1.508 (4)		
			
H1WA—O1W—H1WB	104.5	C14—C13—H13A	109.0
C1—N1—C7	124.4 (2)	C12—C13—H13B	109.0
C1—N1—H1	117.8	C14—C13—H13B	109.0
C7—N1—H1	117.8	H13A—C13—H13B	107.8
C16—N2—H2A	109.5	C10—C9—C8	120.3 (3)
C16—N2—H2B	109.5	C10—C9—H9	119.8
H2A—N2—H2B	109.5	C8—C9—H9	119.8
C16—N2—H2C	109.5	C4—C5—C6	119.8 (3)
H2A—N2—H2C	109.5	C4—C5—H5	120.1
H2B—N2—H2C	109.5	C6—C5—H5	120.1
C15—O3—H3A	109.5	C5—C4—C3	120.0 (3)
C3—C2—C1	122.1 (3)	C5—C4—H4	120.0
C3—C2—Cl1	117.0 (2)	C3—C4—H4	120.0
C1—C2—Cl1	120.9 (2)	C4—C3—C2	119.5 (3)
O2—C14—O1	123.9 (3)	C4—C3—H3	120.2
O2—C14—C13	118.9 (3)	C2—C3—H3	120.2
O1—C14—C13	117.2 (3)	C10—C11—C12	121.4 (3)
C6—C1—N1	121.1 (3)	C10—C11—H11	119.3
C6—C1—C2	115.9 (3)	C12—C11—H11	119.3
N1—C1—C2	122.8 (3)	C9—C10—C11	119.6 (3)
C11—C12—C7	118.5 (3)	C9—C10—H10	120.2
C11—C12—C13	120.4 (3)	C11—C10—H10	120.2
C7—C12—C13	121.1 (3)	N2—C16—C15	110.0 (3)
C8—C7—C12	119.7 (3)	N2—C16—H16A	109.7
C8—C7—N1	121.6 (3)	C15—C16—H16A	109.7
C12—C7—N1	118.7 (3)	N2—C16—H16B	109.7
C9—C8—C7	120.4 (3)	C15—C16—H16B	109.7
C9—C8—H8	119.8	H16A—C16—H16B	108.2
C7—C8—H8	119.8	O3—C15—C16	109.2 (3)
C5—C6—C1	122.5 (3)	O3—C15—H15A	109.8
C5—C6—Cl2	118.4 (2)	C16—C15—H15A	109.8
C1—C6—Cl2	119.1 (2)	O3—C15—H15B	109.8
C12—C13—C14	112.7 (2)	C16—C15—H15B	109.8
C12—C13—H13A	109.0	H15A—C15—H15B	108.3
			
C7—N1—C1—C6	131.3 (3)	C2—C1—C6—Cl2	−176.9 (2)
C7—N1—C1—C2	−52.7 (4)	C11—C12—C13—C14	−100.2 (3)
C3—C2—C1—C6	−4.4 (4)	C7—C12—C13—C14	80.1 (3)
Cl1—C2—C1—C6	174.1 (2)	O2—C14—C13—C12	117.9 (3)
C3—C2—C1—N1	179.4 (3)	O1—C14—C13—C12	−61.2 (4)
Cl1—C2—C1—N1	−2.1 (4)	C7—C8—C9—C10	1.8 (4)
C11—C12—C7—C8	0.9 (4)	C1—C6—C5—C4	0.3 (5)
C13—C12—C7—C8	−179.4 (3)	Cl2—C6—C5—C4	−179.8 (2)
C11—C12—C7—N1	−179.8 (3)	C6—C5—C4—C3	−2.4 (5)
C13—C12—C7—N1	−0.1 (4)	C5—C4—C3—C2	0.9 (5)
C1—N1—C7—C8	−16.6 (4)	C1—C2—C3—C4	2.6 (4)
C1—N1—C7—C12	164.1 (3)	Cl1—C2—C3—C4	−176.0 (2)
C12—C7—C8—C9	−2.0 (4)	C7—C12—C11—C10	0.3 (4)
N1—C7—C8—C9	178.7 (3)	C13—C12—C11—C10	−179.3 (3)
N1—C1—C6—C5	179.3 (3)	C8—C9—C10—C11	−0.5 (5)
C2—C1—C6—C5	3.0 (4)	C12—C11—C10—C9	−0.5 (5)
N1—C1—C6—Cl2	−0.6 (4)	N2—C16—C15—O3	−53.2 (4)

### Hydrogen-bond geometry (Å, º)

**Table:**

*D*—H···*A*	*D*—H	H···*A*	*D*···*A*	*D*—H···*A*
N1—H1···O1	0.86	2.27	2.884 (3)	129
N2—H2*A*···O2^i^	0.89	1.96	2.811 (4)	160
N2—H2*B*···O1*W*^ii^	0.89	2.15	2.947 (4)	148
N2—H2*C*···O1^iii^	0.89	1.92	2.802 (3)	169
O3—H3*A*···O1*W*	0.82	1.96	2.770 (4)	168
O1*W*—H1*WB*···O2	0.85	1.87	2.690 (3)	161
O1*W*—H1*WA*···O1^iv^	0.85	2.00	2.809 (3)	158

Symmetry codes: (i) −*x*, −*y*+1, −*z*+1; (ii) −*x*, *y*−1/2, −*z*+3/2; (iii) *x*, *y*−1, *z*; (iv) *x*, −*y*+3/2, *z*+1/2.

## References

[bb1] Agilent (2014). *CrysAlis PRO*. Agilent Technologies Ltd, Yarnton, England.

[bb2] Castellari, C., Comelli, F. & Ottani, S. (2001). *Acta Cryst.* C**57**, 437–438. 10.1107/s010827010002076x 11313587

[bb3] Etter, M. C. (1990). *Acc. Chem. Res.* **23**, 120–126.

[bb4] Groom, C. R., Bruno, I. J., Lightfoot, M. P. & Ward, S. C. (2016). *Acta Cryst.* B**72**, 171–179. 10.1107/S2052520616003954 PMC4822653 27048719

[bb5] Knaak, J. B., Leung, H. W., Stott, W. T., Busch, J. & Bilsky, J. (1997). *Rev. Environ. Contam. Toxicol.* **149**, 1–86. 10.1007/978-1-4612-2272-9_1 8956558

[bb6] Lemmerer, A., Bourne, S. A., Caira, M. R., Cotton, J., Hendricks, U., Peinke, L. C. & Trollope, L. (2010). *CrystEngComm*, **12**, 3634–3641.

[bb7] Lynch, D. E., Bening, A. S. & Parsons, S. (2003). *Acta Cryst.* E**59**, o1314–o1317.

[bb8] Macrae, C. F., Sovago, I., Cottrell, S. J., Galek, P. T. A., McCabe, P., Pidcock, E., Platings, M., Shields, G. P., Stevens, J. S., Towler, M. & Wood, P. A. (2020). *J. Appl. Cryst.* **53**, 226–235. 10.1107/S1600576719014092 PMC6998782 32047413

[bb9] Moser, P., Sallmann, A. & Wiesenberg, I. (1990). *J. Med. Chem.* **33**, 2358–2368. 10.1021/jm00171a008 2118185

[bb10] Sheldrick, G. M. (2015*a*). *Acta Cryst.* A**71**, 3–8.

[bb11] Sheldrick, G. M. (2015*b*). *Acta Cryst.* C**71**, 3–8.

[bb12] Siemens (1994). *XP*. Siemens Analytical X-Ray Instruments Inc., Madison, Wisconsin, USA.

[bb13] Skoutakis, V. A., Carter, C. A., Mickle, T. R., Smith, V. H., Arkin, C. R., Alissandratos, J. & Petty, D. E. (1988). *Drug Intell. Clin. Pharm.* **22**, 850–859. 10.1177/106002808802201102 3069424

[bb14] Turner, M. J., McKinnon, J. J., Wolff, S. K., Grimwood, D. J., Spackman, P. R., Jayatilaka, D. & Spackman, M. A. (2017). *Crystal Explorer 17.5*. University of Western Australia.

[bb15] Westrip, S. P. (2010). *J. Appl. Cryst.* **43**, 920–925.

[bb16] Wishart, D. S., Tzur, D., Knox, C., Eisner, R., Guo, A. Ch., Young, N., Cheng, D., Jewell, K., Arndt, D., Sawhney, S., Fung, C., Nikolai, L., Lewis, M., Coutouly, M. A., Forsythe, I., Tang, P., Shrivastava, S., Jeroncic, K., Stothard, P., Amegbey, G., Block, D., Hau, D. D., Wagner, J., Miniaci, J., Clements, M., Gebremedhin, M., Guo, N., Zhang, Y., Duggan, G. E., Macinnis, G. D., Weljie, A. M., Dowlatabadi, R., Bamforth, F., Clive, D., Greiner, R., Li, L., Marrie, T., Sykes, B. D., Vogel, H. J. & Querengesser, L. (2007). *Nucleic Acids Res.* **35**, D521–D526. 10.1093/nar/gkl923 PMC1899095 17202168

